# A Review on Genomics APIs

**DOI:** 10.1016/j.csbj.2015.10.004

**Published:** 2015-10-31

**Authors:** Rajeswari Swaminathan, Yungui Huang, Soheil Moosavinasab, Ronald Buckley, Christopher W. Bartlett, Simon M. Lin

**Affiliations:** a Research Information Solutions and Innovation, The Research Institute at Nationwide Children's Hospital, Columbus, OH, USA; b The Battelle Center for Mathematical Medicine, The Research Institute at Nationwide Children's Hospital, Columbus, OH, USA

**Keywords:** Genomics, Data sharing, Application Programming Interface (API), Clinical, Sequencing

## Abstract

The constant improvement and falling prices of whole human genome Next Generation Sequencing (NGS) has resulted in rapid adoption of genomic information at both clinics and research institutions. Considered together, the complexity of genomics data, due to its large volume and diversity along with the need for genomic data sharing, has resulted in the creation of Application Programming Interface (API) for secure, modular, interoperable access to genomic data from different applications, platforms, and even organizations. The Genomics APIs are a set of special protocols that assist software developers in dealing with multiple genomic data sources for building seamless, interoperable applications leading to the advancement of both genomic and clinical research. These APIs help define a standard for retrieval of genomic data from multiple sources as well as to better package genomic information for integration with Electronic Health Records. This review covers three currently available Genomics APIs: a) Google Genomics, b) SMART Genomics, and c) 23andMe. The functionalities, reference implementations (if available) and authentication protocols of each API are reviewed. A comparative analysis of the different features across the three APIs is provided in the Discussion section. Though Genomics APIs are still under active development and have yet to reach widespread adoption, they hold the promise to make building of complicated genomics applications easier with downstream constructive effects on healthcare.

## 1 Introduction

The cost of sequencing an individual whole genome is decreasing rapidly, making broad adoption feasible in both research and clinical care [1]. However, the needs of research and patient care are not completely aligned with regards to genomics. An informed clinical decision for the advancement of healthcare is dependent on research based on sharing of genomic data and clinical information from multiple sources. Also, the emerging practice of personalized medicine requires the integration of genomic data with information in Electronic Health Records (EHRs), which while causing a paradigm shift in the diagnosis and treatment of diseases also introduces greater complexity for data privacy and confidentiality. As research is vital for the future of clinical genomics, the NIH on August 27, 2014, issued the Genomic Data Sharing (GDS) Policy that requires researchers to share any genomic data (human or non-human) with the broader community [2]. Data sharing has been recognized as paramount, not only for the advancement of scientific knowledge but also for the preservation and reuse of information [3], [4]. Beyond the great volume of data that researchers wish to share, there are many different formats, structures, and sources that make data integration a real bottleneck within data sharing protocols.

**Fig. 1:**
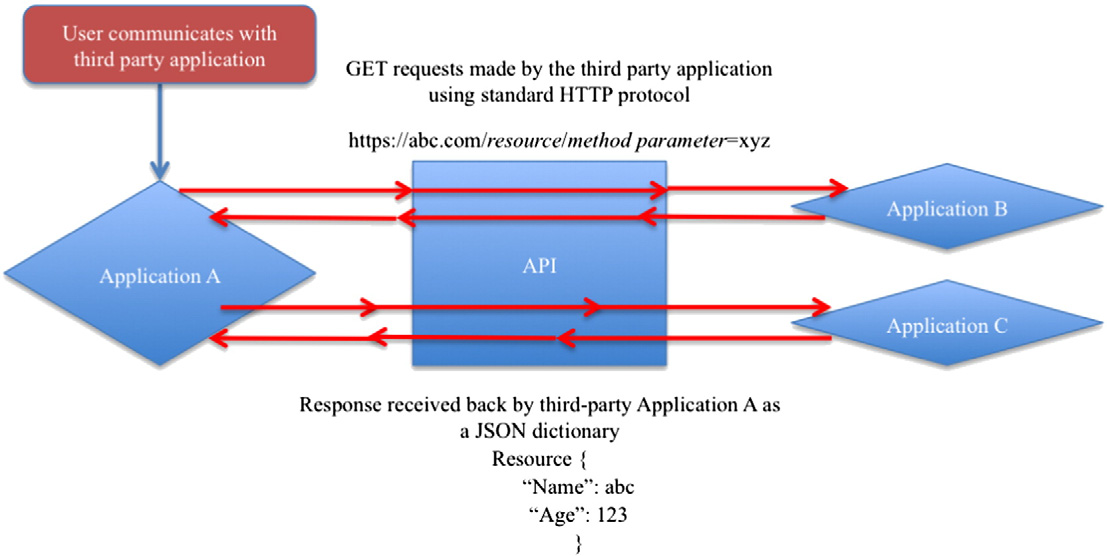
The figure depicts the basic working function of an API. Application A is a third-party application, and Applications B and C are other applications with data-rich backend. A user connects to the third-party application, which then sends in requests, on behalf of the user, to Applications B and/or C and depending on the authentication being successful, receives data back. The figure also shows how a single API can promote interoperability across different data sources, such as B and C.

To meet these rigorous and (somewhat) competing requirements, numerous groups have been working together to create Application Programming Interface (API). In the simplest sense, an API is a set of protocols and instructions that enables one application to communicate directly with another application. These APIs develop a framework of harmonized approaches to enable sharing and integration of data in an effective manner. There are numerous APIs that have been developed for the biology community including, for example, the PubMed API. As shown in Fig. 1, users can connect to any third-party application like Application A, obtain data from Applications B and C through the intermediate API layer and perform improved analysis of all the data within Application A.

API is a mechanism that allows one organization to share data or other resources with the public or a controlled list of individual users. Software developers can make use of APIs to incorporate data into more complex applications. Users benefit from APIs by having access to third-party applications created by the software developers, which provide data from multiple sources. Thus, developers interact directly with the API to apply its rules within their applications; users, on the other hand, run the analysis using the additional data that the developers extract using the APIs. So, in this way, APIs can be thought of as a tool that can promote reuse of the same resources by different applications. Additionally, it is possible to have one common app accessing different applications through their respective APIs to help integrate data from multiple sources. By facilitating data integration functions, APIs help to satisfy the need for data sharing in research.

This review specifically focuses on one kind of API to be used with genomics related applications, termed Genomics APIs. A Genomics API mostly works with some of complex information generated from high throughput experiments. One of the biggest challenges in working with genomics data is the extreme variety of data and an even greater variety of file formats. Every organization, it seems, uses a different constellation of file formats, with further subtle differences arising from the different technologies and platforms that generated the data. As a result, there is no single universally usable tool to access all genomics data across organizations. Development of Genomics APIs is an important milestone toward interoperability in the genomics world. By building a common framework to model the different entities in the genomics and clinical world, the Genomics API can enable effective sharing and integration of data for the advancement of human health.

Though the data integration problem is well defined, the process of putting together a robust, well-defined Genomics API has been a multifaceted challenge [5]. As mentioned previously, genomic data are extremely heterogeneous and come in myriad formats. Genomic data include information within gene expression databases like NCBI GEO, EBI Array Express, as well as sequencing archives such RefSeq and NCBI Sequence Archive. There are a variety of formats and data types, associated with sequencing data. Currently, sequencing platforms output genomic data in either FASTQ, SAM, BAM, or VCF formats, as opposed to the traditional FASTA format alone. Some databases only contain raw read information whereas others can contain detailed information down to the variant level. These data structure inconsistencies are growing along with the emergence of novel genomics technologies, thus making the process of data integration more cumbersome over time. After data integration, another potential problem associated with genomic data sharing is confidentiality. The genome itself can be considered personally identifiable information and simple anonymization techniques are insufficient. Ethical considerations may require special precautions be put in place when dealing with genomic data sharing. This data privacy concern adds an additional layer of complexity when designing a Genomics API to ensure adequate privacy is attained.

Here we investigate some of the major efforts being taken in this field to overcome some of the hurdles mentioned above and put together a framework for a working Genomics API. The review highlights some of the functionalities, reference implementations, and authentication mechanisms for each of the currently available APIs. We provide a comprehensive review to enable researchers and developers to use these API functions when designing similar applications.

## 2 Genomics APIs

For the past 2 years, many groups have been working on creating Genomics APIs that the entire research community can leverage. One of the major efforts in the area came from the Global Alliance for Genomics and Health (GA4GH) consortium.

The GA4GH consortium started in early 2013 and brings together over 200 leading institutions working in healthcare, research, disease advocacy, and information technology with a goal to promote genomic medicine for the advancement of human health [6]. They created coherent teams called “Working Groups,” each tasked with developing products in specific areas, ultimately meeting the global requirement of genomic and clinical data sharing and integration. One of their major goals was the creation of a standard format for representation of genomic data and an interoperable Genomics API built on top of it, which can communicate with genomic data across different sources. The API contains a set of function calls that is used to package genomic information into specific resources and to query different pieces of information. Currently, researchers use the specific APIs offered by the different data hosting repositories for accessing the data. Custom API calls are often written for data stored in a local repository. Even though the data type within all repositories is identical, the data within them is still accessed through multiple different APIs. The framework developed by GA4GH is a prototype to model genomics data coming from various sources such that a single API call can access any of them.

GA4GH does not host any data of its own. To have a working implementation of the above framework, Google decided to work on the project jointly with GA4GH and developed their version of an API that can store and access genomic data from the cloud. Google Genomics is the name of this working product from Google. The first version of Google Genomics API, based on the first version of the GA4GH framework, focused only on storing and accessing genomic sequence reads data. Other organizations like the National Center for Biotechnology Information (NCBI) and the European Bioinformatics Institute (EBI) also implemented this initial version of the GA4GH framework that further demonstrates the interoperability of the framework across different data sources. Of the three organizations mentioned above, Google has been the one actively involved in developing the API and incorporating all changes made on the GA4GH end.

Parallel to this effort, a team from the Harvard Medical Center for Biomedical Informatics was also working on a similar goal of trying to model genomic information in order to fit the clinical realm, specifically on the aspect of integrating genomics data within Electronic Health Records. The SMART Genomics API was developed as a standard of access to genomic data and is the second Genomics API we focus on this review. The project came along as an extension of their SMART clinical initiative that involved developing standards for defining medical information. The SMART Genomics API is built by adding as well as extending existing resources from HL-7, which is a known standard used in clinical settings.

One of the leading personal genomics companies, 23andMe, developed the third API covered in this review, called the 23andMe API. The scope of this API is much more narrow compared with the previous two APIs. The development of this API mainly started due to the interest in making an individual's personal genome data readily accessible to them. Even though the data modeling of the underlying genomic information is slightly different, the end goal is still to be able to share genomic information effectively with multiple third-party applications.

The sections below outline some of the main functionalities and implementation details for each of the APIs mentioned above. All of these APIs are still under active development.

## 3 Google Genomics API

**Fig. 2:**
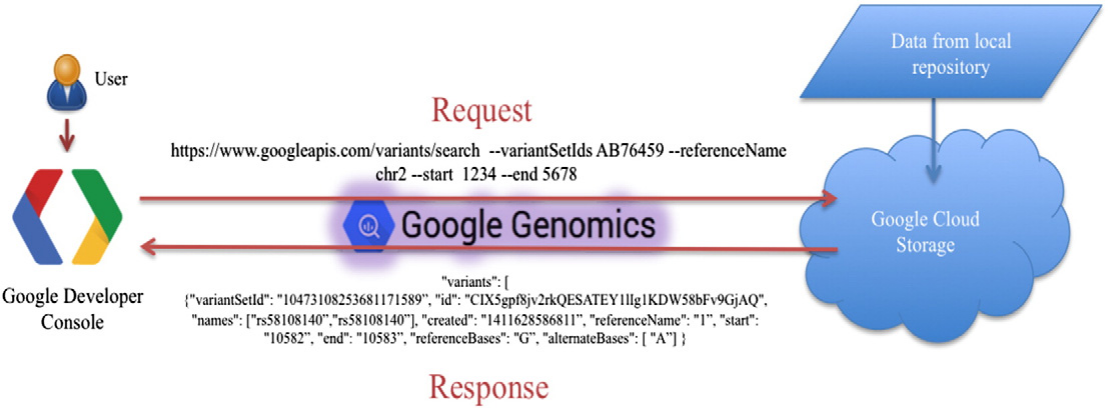
A workflow showing the flow of information through the Google Genomics API. Since there is no real application using the API currently, developers at Google have created a web interface through the Google Developer's Console that allows users to send requests for data access through Google Genomics API. Users log in through the Developer Console and send API request calls to the cloud to fetch information. For example, the figure above depicts an API call for extracting all variants within a given variantSet and chromosomal location. With a successful authentication, Google Cloud will respond back to the user with a JSON dictionary, as shown in the figure.

Google Genomics is an API for storing, processing, exploring, and sharing genomic data [7]. This API is the search giant's first product for the genomics world that utilizes its cloud computing service. Google is one of the many actively participating organizations within the GA4GH working groups. The implementation of Google Genomics is one of the first efforts in bringing the vision of GA4GH into fruition. Along with implementing the framework supported by GA4GH, Google Genomics also supports access to other features by taking advantage of the humongous storage and computational capacity available within Google Cloud. The most recent version of Google Genomics (v2.0) is based on the current version of the GA4GH framework (v0.5). All data accessed by the API are stored within Google Cloud Storage. Anyone with access to Google Cloud Storage is entitled to use the Google Genomics API, making a large user community for it. There are currently no real-world applications that use the API. However, Google provides researchers with a platform to execute sample API calls on the publicly available 1000 Genomes dataset through the Google Developer Console. Complete details on the API and its implementation can be obtained through the Google Genomics website.^1^ An example request response workflow using Google Genomics is shown below in Fig. 2.

**Fig. 3:**
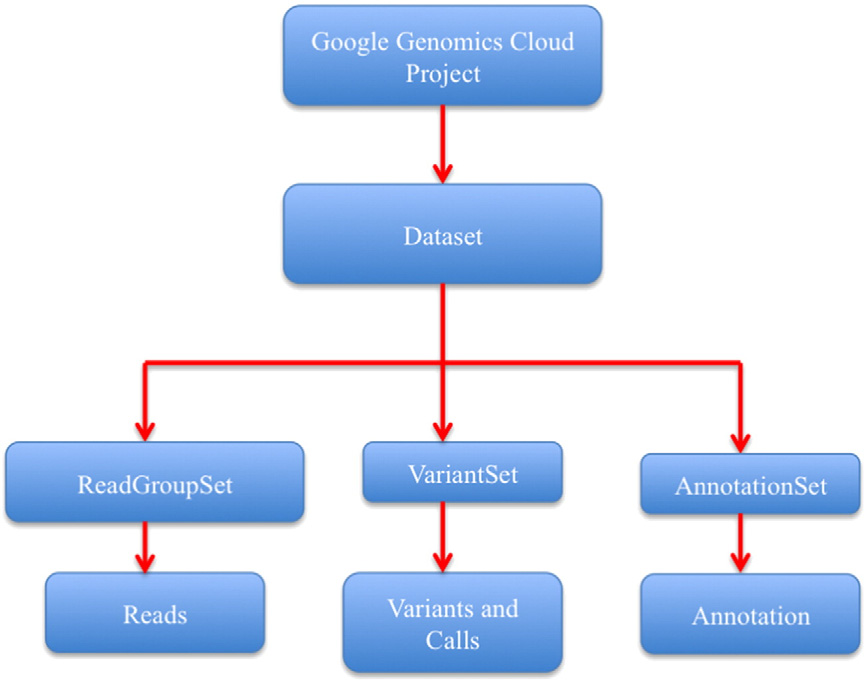
An overview of the Google Genomics Data Model showcasing the key resources and their relationship to one another.

The Google Genomics API has methods that can work with both read and variant data as input. The data accessed by the Google Genomics API need to be first imported into Google Cloud Storage from any local repository and then loaded as a dataset into the Genomics API. A dataset is a logical grouping of read, variant, and annotation data for any given project, as explained in Fig. 3. Users can set access permissions at the dataset level to either make the data publicly available (for example, the 1000Genomes dataset) or provide restricted access to authorized users only.

The Google Genomics community has created several client-side tools, along with the Developer Console for users to interact with the API and fully explore its functional capabilities. A list of the different options is available through the Google Genomics GitHub page [8]. Many of them are command line clients written in different languages like Java, Python, Ruby, etc. that users can utilize to interact easily with the API. Google also offers a more interactive way of exploring the API using the Google Developers Console. This console provides a web interface to experiment with managing users, authorization information as well as execute different queries using the publicly available 1000Genomes Dataset.

The Google Genomics API provides the standard methods like create, delete, search, update, etc. for accessing each of the resources mentioned in Fig. 3 through standard HTTP request calls of POST, DELETE, GET, and PUT. Both aligned and unaligned reads can be imported into Google Genomics API in BAM and FASTQ formats, respectively. Variant data can be imported directly from VCF files. One of the key features of Google Genomics API is the extraction of slices of genomic data (either alignments or variants) and outputting them as a JSON dictionary to the user. The fields contained in the dictionary can be formatted according to the requirements of the user. Below are some example API calls using Google Genomics for specific use cases.

⦁ Search for all variants in a variant Set and within a given genomic range of coordinates.
  ○ Request:
    POST https://www.googleapis.com/genomics/v1beta2/variants/search --*variantSetIds* 10473108253681171589 --*referenceName* 1 --*start* 1234 –*end* 5678
  ○ Response:
    {"variantSetId":"10473108253681171589","id":"CIX5gpf8jv2rkQESATEY1lIg1KDW58bFv9GjAQ","names": ["rs58108140","rs58108140"],"created": "1411628586811","referenceName": "1","start": "10582","end": "10583","referenceBases": "G","alternateBases": ["A"],"quality": 100,"filter": ["PASS"]}
⦁ Search for all Call Sets within a given Variant Set.
  ○ Request:
    POST https://www.googleapis.com/genomics/v1beta2/callsets/search --*variantSetIds* 10473108253681171589
  ○ Response:
    "callSets": [{"id": "10473108253681171589-0","name": "HG00261","sampleId": "HG00261","variantSetIds": ["10473108253681171589"],"created": "1411600138221","info": {}}
⦁ Extract all variant Set's within a given dataset.
  ○ Request:
    POST https://www.googleapis.com/genomics/v1beta2/variantsets/search --*datasetId* 10473108253681171589
  ○ Response:
    "variantSets": [{"datasetId": "10473108253681171589","id": "10473108253681171589","referenceBounds": [{"referenceName": "1","upperBound": "249881990"}

The response time for executing the above-mentioned queries is relatively quick. Such a design is very well suited to a clinical environment where low response time is a key requirement.

For some of the more computationally intense queries, such as calculating allele frequency over a set of variant calls or running a genome-wide association analysis, data can be exported from Google Genomics into Big Query, Google's Big Data Analytics platform, and then executed. This is Google's distributed computing environment that uses SQL-like functions to query some of the massive datasets and execute some of the computationally demanding queries.

At present, Google charges for data storage in the cloud and also for the API calls made to access data. As an incentive, Google does supply a fixed set of free resources, defined by a set of quotas [9]. Google Genomics is an actively developing project with a growing user community. Being a member of the GA4GH consortium, the group at Google will be actively pushing all changes made to the GA4GH framework, into their system. At the same time, there are a lot of upcoming genomic projects that will also be taking advantage of the Google Cloud to store their data. More functions will be developed in the future within the Google Genomics API for users and developers to explore these datasets as well.

## 4 SMART Genomics API

SMART stands for Substitutable Medical Applications, Reusable Technologies. The two keywords here are Substitutable and Reusable. The principle of substitutability allows for different apps to be built on top of a healthcare system where one app can be readily substituted for another, thus eliminating the barrier to software programming [10]. Reusability, on the other hand, allows the same app to work across different healthcare systems like Electronic Health Record (EHR) and Personal Child Health Record (PCHR). To achieve this substitutability and reusability, an API needs to be defined with a common vocabulary that can be used to interact with data from different sources. Creation of APIs also makes the underlying data reusable by different applications. The SMART platform was first implemented in the healthcare domain. The product created was the SMART Clinical API [11]. The idea behind this initiative was to have a common platform such as EHR or PCHR and build multiple core applications that use its data. The applications use some of the resources defined by the SMART Clinical API to access information from multiple different health IT systems. This API was an initiative by a group at Harvard Medical School. Inspired by the success of the Clinical API and given the emerging need for integrating genomic information into EHRs by point-of-care providers, a group at the Harvard Medical Center for Biomedical Informatics decided to extend the functionality of the SMART platform to access genomic information. This marked the beginning of the SMART Genomics API journey. The project started around the middle of 2013, almost in parallel to the GA4GH initiative. The group at Harvard is also part of the GA4GH consortium and the SMART initiative is in perfect line with their goal of bringing together clinical and genomic information. SMART Genomics uses the Fast Health Interoperability Resource (FHIR) as its base framework [10]. The Genomics API is built by extending some of the existing resources within the Clinical API to fit the needs of the genomics community. Detailed information regarding the different SMART Genomics API resources can be obtained through their website.^2^

**Fig. 4:**
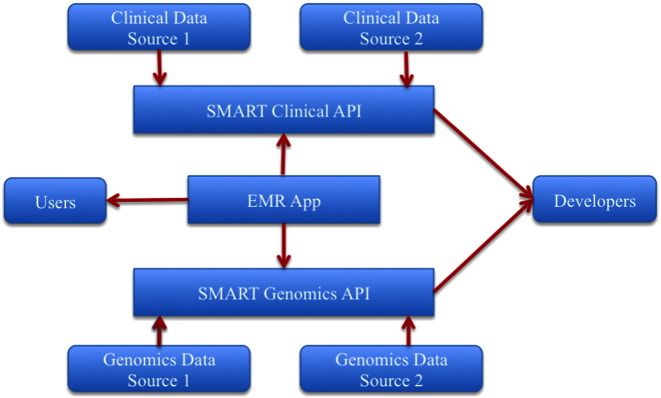
Schematic representation of the SMART containers and how both the SMART Genomics API as well as the SMART Clinical API can communicate through the common EMR App (Electronic Medical Record) by accessing data from different sources.

There are three main entities involved within a SMART model: data sources, developers, and users, as shown in Fig. 4. Data sources are defined as the origin from where the data are coming. The aim is to create an API that is interoperable across different sources. The sources can be clinical sources such as different EHRs as well as genomic data sources. The developers extract these data, through the API and plug it into their apps to perform various analyses. The users are the ones who make use of the apps to get meaning out of the data. In the SMART clinical world, data sources such as health IT systems are named “Containers.” Examples of data sources are data coming from EHRs, PCHRs, etc. The API defines distinct resources to access data from these Containers.

The SMART Containers can store both Clinical and Genomics data from different sources and they are all connected to a common EHR Application through the SMART API calls (Fig. 3). Again, the API is developed to be EHR agnostic. This helps a physician correlate between clinical and genomic data and decide on the appropriate course of treatment.

There are three major resources within the SMART Genomics API: *Sequence*, *Sequencing Lab*, and *Genetic Observation.* Below is a brief overview of each of the three resources [12].

*Sequence*: This resource is used to store sequence data. The data can be DNA, RNA, or amino acid information, in varied formats (like VCF, GFF, etc.). The resource is created in an extremely abstract manner that is agnostic to differences in file formats. Some of the information encoded by this resource includes read of the sequence, type of sequence, chromosomal location of sequence, etc.
*Sequencing Lab*: This is an extension to the Procedure resource within HL-7 FHIR. As this resource records all details on the different procedures performed on a patient, Sequencing Lab stores results on all the sequencing reactions and data pre-processing that were performed on the patient's genome/exome/proteome. This resource helps link patient sequences to the original source.
*Genetic Observation:* This is an extension to the existing Observation resource in HL-7 FHIR. As this records details on any observation (for example, blood pressure level) related to a patient, Genetic Observation is used to store and extract genetic/genotype-related changes on a patient. This resource links a patient's genotype to phenotype.

All three resources are related to one another. For instance, the Genetic Observation resource is linked to the Sequence resource since it contains “Source of sequence” as one of its elements.

For a developer or any third-party application using the API, the input data source is health information from EHRs as well as genomic sequence data. The exact format for the input sequence data is not specified clearly anywhere in the API documentation.

Since the data source for the SMART APIs (both clinical and genomic data) is the secure health record systems, the user base for the API is not as broad as the Google Genomics API but is limited to only those users having access to the health record systems. Currently, there are no reference apps that use the API to access any specific data source. However, the group at Harvard does provide a demo app to demonstrate the functionality of the API as well as how to perform API requests. Information on setting up this demo server and data sources can be obtained here.^3^

The methods used by all resources in the SMART Genomics API are Get, Create, Update, and Search that are transformed into standard HTTP requests of GET, POST, and PUT calls. The user can specify to receive a response back in either JSON or XML formats by appending?_format={xml|json} to the HTTP requests. Some sample API calls that can be executed by the SMART system are the following:

⦁ For a given patient and sample type of prenatal, extract the sequence information within the given range of coordinates.
  ○ GET/Sequence? patient=123&sample=prenatal&coordinates=chr3:3306-3307
⦁ Extract results of a sequencing experiment performed on a patient on a given date.
  ○ GET/Procedure?patient=123&Date=05/05/05
⦁ Create a record containing the Genetic Observation associated with a patient.
  ○ POST/Observation?patient=123
⦁ Extract the genetic observations (variations) for a given patient associated with a specific condition.
  ○ GET/Observation?patient=123&AssessedCondition=Diabetes

The documentation for the SMART Genomics API does not provide examples of the JSON response returned back to the user. It is clearly visible from the above use cases that the API can interact with both clinical and genomic information at the level of a single patient. Offering clinical and genomic information jointly is an ideal scenario for a physician who at any given time point tries to correlate between the genomic and medical data for a particular patient.

There are a few existing applications that currently use the SMART Genomics API. One such application is the Genomics Advisor. This is one of the initial applications to make use of the SMART Genomics API in order to integrate clinical and genomic information to create a patient-centric health and genomics record. This app helps physicians assess a patient's risk toward different diseases like diabetes using both their genomic and clinical information. This app also supports the integration of consumer-based genomics information from companies such as 23andMe. Another application, Variant Mapper, compares patient's DNA variants to a database of genotype–phenotype associations (such as ClinVar) and alerts the physician about potential risk factors for the patient. Each of these tools – Genomics Advisor and Variant Mapper – can be loaded as individual apps on top of any given Health IT system.

The future for the use of SMART Genomics API looks bright as newer apps are being developed using the APIs functionality.

## 5 23andMe API

Along with academic labs and research institutions, commercial organizations are also working to make genetic data readily available to patients through third-party client applications in a seamless and secure manner. Some of these applications can become great tools for patients in helping them understand their genetic data through a more visual and graphical representation. One such organization that decided to make broad use of its rich data source for the benefit of its customers is the privately held personal genomics company named 23andMe. The product designed by them is termed the 23andMe API. The company currently does not provide a detailed analysis of a customer's genetic data. APIs such as these empower all the customers who have their genome sequenced through 23andMe to gain a better understanding of their body functions through secure interaction with multiple client applications. Detailed information on the API can be obtained through the 23andMe website.^4^

Compared with the previous two APIs, the user community for the applications that access this API is the small subset of 23andMe customers. The API also defines a set of resources with specific endpoints of data access. The different resources in the API are User, Ancestry, Genetics, Health, and Phenotype. The endpoints describe the specific pieces of information within the resource that the API is trying to access. For example, the Genetics resource can have two different endpoints, genotypes, or genome. When the genotypes endpoint is specified, the API returns the genotypes in a patient at a given genomic location, whereas with genomes as the endpoint, the entire genome for the specified patient is returned as a response. The API specializes in defining multiple endpoints for the Ancestry and Health Risk resources. There is no documentation defining any relationship between the different resources.

**Fig. 5:**
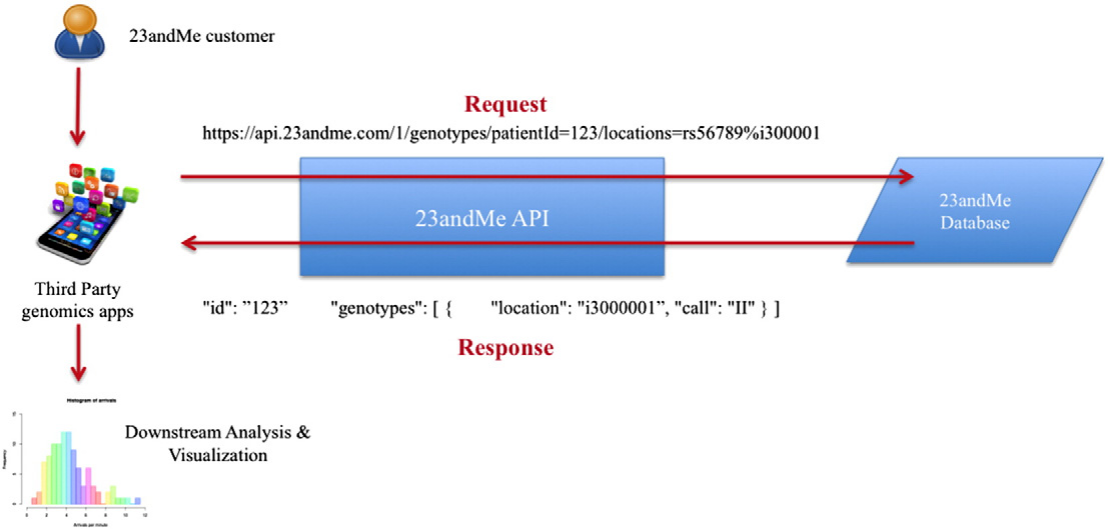
Workflow for the 23andMe API. A 23andMe customer, through a third-party application, sends API calls requesting for their genotypes within a given chromosomal location. This information is present within the 23andMe database as the customer's genetic test analysis report. Once the third-party application is successfully authenticated, the appropriate response is sent back to the customer. This response can then be combined with other sources of information for a more detailed downstream analysis and visualization.

The input data that the API tries to access are the genome sequence, health, ancestry, and user information of their customers, directly from 23andMe database. Data are returned to users as a standard JSON dictionary. Fig. 5 depicts the flow of information through the 23andMe API.

Unlike the other two APIs, there is really no easy way to create a reference implementation for the 23andMe API since the data are securely managed within 23andMe's database and the only people with access to it are personnel within that organization. However, their documentation does contain an API reference page that goes over some of the details on using the API. Below are shown a few examples of some of the different use cases for the 23andMe API:

⦁ Extract the genotypes for a patient at a given location.
  ○ Request:
    GET https://api.23andme.com/1/genotypes/patientId=123/locations=rs56789%i300001
  ○ Response:
    "id": "c4480ba411939067"
      "genotypes": [{"location": "i3000001", "call": "II"}
⦁ Extract the value for a given phenotype in a given patient.
  ○ Request:
    GET https://api.23andme.com/1/phenotypes/patientId=123/phenotype=gender
  ○ Response:
    "phenotype_id": "sex",
    "value": "male",
  ○ There are a very limited set of phenotypes (mostly related to patient demographics) that can be obtained through this API call.
⦁ Obtain all risks associated with a particular patient.
  ○ Request:
    GET https://api.23andme.com/1/risks/patientId=123
  ○ Response:
    "id": "c4480ba411939067",
    "risks": [
      {"description": "Atrial Fibrillation",
      "report_id": "atrialfib",
      "population_risk": 0.2715,
      "risk": 0.4164
      }
  ○ There is a list of diseases for which risk analysis is done at 23andMe and the response to this call returns both the individual's risk as well as the population level risk for every disease.

Despite the limited use cases and user community associated with the 23andMe API, there is utility for this API. The application of personalized medicine necessitates actual involvement of the patients themselves as part of the total care process, and this is being achieved through the creation of the 23andMe API.

## 6 Genomics API Authentication

One of the most important considerations while developing an API is to provide the appropriate mechanism to protect users' confidential information. With genomics APIs, this becomes an absolute necessity since the genome is a person's blueprint to all his health-related information. API service providers have been using various security protocols that have evolved over the past years, each with their own set of pros and cons. All of the genomics APIs mentioned in this review use the OAuth 2.0 protocol for authenticating and authorizing requests. The OAuth protocol authenticates an application using a secure token-based approach rather than have a user provide their password to every application using the API. One of the biggest advantages of this approach is that it prevents a user's password from propagating through various third-party applications. There are 2 different levels of authentication supported by OAuth, one that requires users to explicitly sign in through a username and password and the other that simply provides an API key to any user requesting access to the application. Since both SMART Genomics and the 23andMe API deal with patient information, their authentication requires users explicitly to validate any third-party application requesting their data. Google Genomics also use this approach when dealing with private data on the cloud. For public data, an API key works just as fine. The main advantage of using an OAuth protocol is it protects a user's username and password from directly being shared across the Internet and with millions of third-party applications. The downside is the complexity of the protocol that can make it slightly cumbersome for developers to incorporate it within their application. Some versions of OAuth, for instance, the OAuth 2.0 used by the Genomics API teams, also requires *https* for all purposes of data transfer, thus providing further encryption and security.

## 7 Discussion

The main focus of this review has been to address the need for genomic data sharing and data integration to facilitate improvement of healthcare practice. Even though the implementations and functionalities of all the three Genomics APIs – discussed in the previous section – are slightly different, they are all working to meet a common end goal. For instance, the current methods enabled within Google Genomics API do not provide any means of accessing clinical information. The input data are limited to sequencing information in the form of reads, variants, and annotation. On the contrary, both the SMART API as well as the 23andMe API can access both types of information for any given patient. That being said, Google has plans to incorporate this functionality to their existing system in future versions. The fact that Google Genomics API accesses data directly from the cloud adds an additional layer of complexity to extend the current system to access clinical information. Table 1 below tries to provide a comparative view across the three Genomics APIs for a list of different features. The table can help researchers have a quick grasp of the strengths and weaknesses of the different APIs and guide them to choose the most appropriate one for their use case.

We can clearly see from the above table that most of the features are available across all three APIs but implemented in various ways. On the other hand, there are also some features that are unique to only a few of them. The range search functionality is available with both the Google Genomics and SMART Genomics APIs, but missing from the 23andMe API. This can be attributed to the differences in user base for the different APIs. The 23andMe data are only accessible to applications that can be authorized by a 23andMe customer, most of whom have no requirement to perform a range search to look for a list of variants. Their main focus is to look for individual variants and assess their risk for certain diseases. Thus, the requirement of the user community is what dictates the functionality of the API.

The major goal of all the APIs discussed in this review has been to encourage and facilitate sharing and integration of genomic and clinical data. We would like to point out that we do not intend to provide any biased opinions through this review regarding which API to use. The ideal choice of API is entirely based on the requirements of the user community and the research question that is trying to be answered. Moreover, most of the genomic APIs are still under active development; the syntax, scope, and functionality might change over time.

## Author Contributions

RS analyzed different genomic APIs, assessed their functionalities, and wrote the draft. YH, RS, and SML contributed to the conception and designed the study. RS, YH, SM, RB, CWB, and SML wrote and revised the article.

## Figures and Tables

**Table 1:** A comparative view across the three Genomics APIs for a list of features.

Features	Google Genomics	SMART Genomics	23andMe
*Input data to API*	Currently, limited to only sequencing information in the form of reads, variants, and annotation	Some of the Genomics resources are extensions of previously existing Clinical resources	Has capability to use both genomics and clinical resources
*Location of data*	Data need to reside within Google Cloud Storage	Data available within EHR's and other genomics data sources	Data from 23andMe database
*API response*	Returns only JSON formatted response	Returns either JSON or XML formatted response	Returns JSON formatted response
*Ability to import data*	Can import both reads and variant data from BAM and VCF files	Create call available for certain resources	API only used for data retrieval through GET calls
*Range search for variants in a given individual*	Available	Available	Not available
*Identify risk for a disease in an individual*	Not available	Available	Available
*Availability of reference applications using the API*	Client libraries and interactive API Explorer through Google Console	Some applications like Genomics Advisor, Variant Mapper currently use the API	Not available
*Authentication*	Uses OAuth2.0	Uses OAuth2.0	Uses OAuth2.0

## Footnotes

1 https://cloud.google.com/genomics/what-is-google-genomics.

2 http://projects.iq.harvard.edu/smartgenomics.

3 https://gist.github.com/DSrcl/0f2edcd8cd1cad2775e9.

4 https://api.23andme.com/overview/.
